# Clinical Decision-Making During Psychiatric Ward Rounds

**DOI:** 10.3389/fpsyt.2021.721699

**Published:** 2021-09-13

**Authors:** Fabian Holzhüter, Florian Schuster, Stephan Heres, Johannes Hamann

**Affiliations:** ^1^Klinik und Poliklinik für Psychiatrie und Psychotherapie, Klinikum rechts der Isar, Technische Universität München, Munich, Germany; ^2^kbo-Isar-Amper-Klinikum München-Nord, Munich, Germany

**Keywords:** shared decision making, ward round communication, patient-doctor communication, patient participation, psychiatric in-patient

## Abstract

**Introduction:** For psychiatric in-patients, ward rounds are a prominent occasion for decision making. As previous findings on shared decision-making (SDM) patterns mostly derive from out-patients and one-to-one-consultations, it was our aim to investigate SDM during psychiatric ward rounds.

**Methods:** We conducted a cross-sectional study and included *n* = 62 in-patients from seven different psychiatric wards. We collected data from the patient and the treating physician before and after ward rounds and recorded the interaction.

**Results:** We identified two groups of patients regarding their attitude toward ward rounds (no expectations vs. clear agenda). The latter showed higher active engagement, expected more decisions to be made and discussed more topics. Generally, observer rated SDM was low, with vast differences between the doctors' and the patients' perception.

**Conclusion:** Doctors and patients perceive ward rounds differently and there is a discrepancy between subjective and objective involvement. A rather paternalistic doctor-patient-relationship is observed, while patients feel sufficiently involved and vastly satisfied. The potential of ward rounds maximizes if patients have an agenda. Consequently, motivating patients to prepare themselves toward ward rounds should be part of the weekly routine, as well as improving patient participation and information procedures during ward rounds.

## Introduction

Shared decision making (SDM) has made its foray into mental healthcare in recent years (1). Besides ethical considerations, it is the hope for improvements in patient care that facilitates the investigation and implementation of SDM (2). In fact, recent findings on SDM in mental health settings underpin this assumption with variables such as treatment satisfaction, therapeutic alliance or treatment adherence being improved (3, 4).

Patient-clinician-communication is at the core of SDM and has mostly been studied in depth in somatic medicine (5) and mental health outpatient care (6, 7). Research on SDM for in-patients in psychiatric hospitals is few (8, 9) and it has to deal with some special features of clinical decision making. In this setting, it is not a patient-physician-dyad that makes therapeutic decisions. Rather, patients are faced with multi-professional teams, consisting of several physicians (e.g., residents and consultants) as well as other professional groups including nurses, social workers and psychologists. As a result, in contrast to outpatient treatment, decision making does not solely take place during a definable consultation, but rather in various circumstances (e.g., team meeting, advice from the consultant etc.).

Probably the most prominent occasion for decision making in psychiatric in-patient treatment is the (consultant) ward round, which regularly takes place in most psychiatric departments (10). Usually these ward rounds are scheduled on a weekly basis, involve several stakeholder groups on the ward (consultant, residents, nursing team, social worker, other stakeholders), and serve as the focal point for clinical decision making.

While ward rounds are undoubtedly helpful in integrating views of different team members and fulfilling teaching obligations (11), patients often feel unheard, as though important information is being withheld (12, 13), and sometimes do not feel involved at all (14). These findings may be caused by the unique features of consultant ward rounds such as time pressure or patients being confronted with many different team members, and thus, having to negotiate with a majority of professionals that are sometimes perceived as hostile.

As previous findings on shared decision-making patterns mostly derive from one-to-one-consultations and are therefore not directly transferable to ward rounds settings, it was the aim of the present explanatory study to investigate clinical decision making and patient participation during psychiatric ward rounds.

## Methods

We conducted a cross-sectional, non-interventional study on consultant wards rounds including in-patients from 7 different general psychiatric wards of 2 different hospitals, 3 of wards were open, 1 closed, and 3 optionally closed. Data collection took place in September and August 2019. We approached patients and their clinicians 1 day prior to the ward round. We informed the patients about the study and explained to them that participation was voluntary. We were present during the consultation and we met them again shortly after the ward round had taken place. All patients being treated on the respective ward during our study visit were eligible for participation and were asked to provide written informed consent. Since we used questionnaires in German language, exclusion criteria were lacking knowledge of German language and inability to provide written informed consent.

After patient's informed consent, but prior to ward rounds, the treating physicians gave information about diagnoses, duration and severity of the illness using Clinical Global Impression (CGI) and Global Assessment of Functioning (GAF) scores (15, 16). Patients answered questions about their socio-demographic background, their expectations and wishes concerning the upcoming round (with a focus on whether they had an agenda with regard to the ward round). All patients additionally completed the following questionnaires regarding their experiences during hospital admission [MacArthur Admission Experience Survey (17)], their preferences for participation in decision making [Autonomy Preference Index, API, (18)] and their active engagement in decision making [PatPart-19 (19)].

During ward rounds, we audio-recorded the conversation between the attending physicians (and other team members) and the patient. We measured the duration of interaction between the patient and the health care team and assessed the number of team members actively taking part in the consultation. From the audiotapes, we rated the consultation by using the observer OPTION-Scale (20), a validated rating tool for assessing the amount of shared decision making during consultations.

After the ward round, the consultant physician was requested to rate on five-point Likert scales who was making decisions and to what extent patients were involved in the process of SDM. Likewise, patients were approached directly after the ward round (or the day after, if patients were not accessible) and asked about how they perceived the ward round. This included questions on their satisfaction with the ward round and their perceived role in the decision process, including the SDM-Q-9-questionnaire (21).

Data was analyzed using SPSS (Version 25 IBM Corporation 1989, 2017). We used descriptive statistics, *t*-tests and chi-square tests. The authors have used the mean and standard deviation for rating scales and percentages for categorical data. Special focus was put on group comparisons between patients who entered the ward round with a clear agenda vs. those who did not. We analyzed correlations between all measures. Two raters assessed the OPTION scale and the categorization of the topics mentioned. The inter-rater-reliability using Cohen's Kappa was ≥0.82 for every item. For the statistical analyses, we focused on the two categories with the most patients (i.e., “no expectations” and “agenda,” see below).

The Ethics Committee of TU Munich approved the study protocol.

## Results

### Sample

One hundred and thirty-eight patients were approached and 62 agreed to participate. The most common reason to deny participation was skepticism toward medical investigations in *n* = 37 cases, followed by skepticism toward the recording in *n* = 26 cases. Most of these patients were female and diagnosed with psychotic disorders. The final study population consisted of 61% (*n* = 38) female and 39% (*n* = 24) male patients. The mean age was 45 years (±16 SD; Min-Max: 18–78). 65% of the patients were treated on open wards and 35% on locked wards. The most frequent diagnoses were mood disorders (42%, *n* = 26) and psychotic disorders (35%, *n* = 22) (Table 1).

**Table 1:** Patient characteristics.

Age	Mean: 45 years (±16 SD; Min-Max: 18–78)	
Sex	Female	61% (38/62)
	Male	39% (24/62)
Diagnoses (ICD-10)	F0 Organic mental disorders	3% (2/62)
	F2 Schizophrenia, schizotypal and delusional disorders	35% (22/62)
	F3 Mood disorders	42% (26/62)
	F4 Neurotic, stress-related and somatoform disorders	10% (6/62)
	F6 Disorders of adult personality and behavior	10% (6/62)
Duration of illness	Mean: 13 years (±11 SD; Min-Max: 0–45)	
Total amount of stays in hospital	Mean: 7 (±8 SD; Min-Max: 1–43)	
Type of ward	Open ward	65% (40/62)
	Closed ward	35% (22/62)

### Patients' Expectations Before the Ward Round

Patients' answers regarding their expectations toward the upcoming ward round were categorized into four groups. The majority of patients had either no expectations regarding the ward round at all (*n* = 26, 42%, “no expectation”), or a clear agenda (*n* = 25, 40%, “agenda”). Ten percentage of the patients (*n* = 6) sought information exchange without a clear agenda and 8% (*n* = 5) had negative expectations regarding the ward round (i.e., anxiety, feeling judged, feeling unseen). Patients' participation preferences (API-scores) were in the medium range of possible values (mean = 46 of 100 points, ±22 SD; Min-Max: 0–94), with no statistical difference between the two groups (“no expectations” vs. “clear agenda”). Patients' self-rated active engagement in decision making (PatPart19) was in the medium to upper range of the scale (mean = 65 of 100 points, ±14 SD; Min-Max: 36–97) with patients who had “no expectations” (mean 60 ± 14) expressing lower scores than patients with an “agenda” (mean: 69 ± 15 SD; *p* = 0.03). Approximately half of the patients (47%) were expecting decisions to be made, with a significant higher number in the “agenda” group (*p* = 0.03). Generally, patients with an “agenda” planned to discuss more topics than patients with “no expectation” (*p* = 0.03) and more frequently anticipated the ward round to serve as an opportunity to make decisions (*p* = 0.02; see Table 2).

**Table 2:** Main results concerning patient participation.

	**Agenda**	**No agenda**	
Active engagement in decision making (PatPart-19, Max = 100)	Mean: 69.0 ± 15.5 SD	Mean: 59.9 ± 13.6 SD	*p* = 0.03
Autonomy preference index (Max = 20)	Mean: 10.6 ± 4.1 SD	Mean: 12.0 ± 3.1 SD	*p* = 0.16
Number of topics to discuss	Mean: 3 ± 1 SD	Mean: 2 ± 1 SD	*p* = 0.03
“Do you think that decisions will be made during this ward round?”	Yes 72% (18/25) No 20% (5/25) I don't know 8% (2/25)	Yes 27% (7/26) No 69% (18/26) I don't know 4% (1/26)	*p* = 0.02
Observer-rated amount of shared decision making during consultation (OPTION scale, Max = 48)	Mean: 13 (±7 SD; 3–25)	Mean: 14 (±4 SD; 6–21)	*p* = 0.07
Perceived role in decision process (SDM-Q-9 questionnaire, Max = 100)	Mean: 49.9 ± 24.3 SD	Mean: 48.5 ± 24.0 SD	*p* = 0.84
Satisfaction with ward rounds	Not to moderately satisfied 24% (6/25)	Not to moderately satisfied 31% (8/26)	*p* = 0.4
	(very) satisfied 76% (19/25)	(very) satisfied 69% (18/26)	
Importance of ward rounds	Very important 42% (8/19)	Very important 9% (2/23)	*p* = 0.04
	Important 42% (8/19)	Important 52% (12/23)	
	Moderate 11% (2/19)	Moderate 4% (1/23)	
	Unimportant 5% (1/19)	Unimportant 9% (2/23)	
	Very unimportant 0% (0/19)	Very unimportant 26% (6/23)	

Interestingly, patients within the “agenda” group had significantly more often restrictions to leave the ward [36% (9/25) vs. 4% (1/26)].

### Decision Making Patterns During Ward Round

All ward rounds began with a clinical discussion of all patient cases between the consultant and other team members, before the actual consultations took place. In all ward rounds, patients then entered a meeting room and were awaited by the clinical team. Mean duration of the conversation between ward staff and patients was 6 min 25 s (median: 5 min 20 s). The mean number of people who were present during the conversation was 6.4 (±1.7 SD, Min-Max: 4–9, Median: 6; i.e., patient, attending physician, residents, nurses, social workers, psychologists, occupational therapists), whereas the mean number of people actively taking part in the consultation was 3.2 (±0.9 SD, Min-Max: 2–6, Median: 3).

Overall SDM-rating with the OPTION-scale was rather low (mean: 14 of 48 ± 5 SD, Min-Max: 3–25). We found no differences in the OPTION sum scores between the groups with an “agenda” or with “no expectations” (Table 2). There was no correlation between OPTION sum scores, participation preferences (API) or patients' active engagement in decision making (PatPart19).

In addition, we categorized the (medical) topics discussed into 10 possible categories. Of which, the three most crucial categories (“medication,” “discharge,” “permission to leave”) were defined as “main categories” and the other seven as “additional categories” (“patient's current condition,” “other therapies” (i.e., psychotherapy, occupational therapy, art therapy, etc.), “legal support/work,” “diagnostics/somatic health,” “compulsory measures,” “private life/relations,” and “follow-up care”). Concerning the three main categories, for every patient we documented whether a decision was actually made and by whom this decision was made (on a scale from 1 = only by the patient to 5 = only by the doctor). The topic discussed most frequently was the “patient's current condition” (*n* = 51), followed by “other therapies” (*n* = 35), “legal support/work” (*n* = 33) and “medication” (*n* = 32). Most commonly, the attending physician initiated the discussion of those topics (mean = 2.4 ± 1.4 SD, Min-Max: 0–6 per conversation), followed by the patient (mean = 1.9 ± 1.6, Min-Max: 0–6 SD per conversation). The resident or other participants in the ward round contributed far less frequently.

Generally, more decisions were made than patients originally expected (73 vs. 47% of the consultations). According to our observer rating, doctors mostly (46%) or only (10%) made decisions during ward rounds and mutual decision making occurred in 33% of the cases.

### Patients' and Physicians' Perceptions of Decision Making During the Ward Round

After the ward round, doctors mostly stated that they were mainly responsible for the decisions made. In contrary, patients mostly reported mutual decision making. This trend also occurred when being asked about involvement in the process of decision. Patients mostly saw themselves “much” involved (45%), while doctors mostly reported “moderate” involvement (44%) In both cases, there was a very poor agreement between doctors and patients (κ = −0.08, −0.04; see Table 3).

**Table 3:** Doctors' and patients' perception of decision making during ward rounds.

**Who made important decisions during the conversation?**			**Doctors**		**Total**
			**Patient participation in decision making**	**No or little participation**	
	Patients	Patient participation in decision making	31% (19/62)	29% (18/62)	60% (37/62)
		No or little participation	24% (15/62)	16% (10/62)	40% (25/62)
	Total		55% (34/62)	45% (28/62)	100% (62/62)
	κ = −0.08				
**How much was the patient involved in decision making?**			**Doctors**		**Total**
			**Low**	**High**	
	Patients	Low	2% (1/62)	11% (7/62)	13% (8/62)
		High	14% (9/62)	73% (45/62)	87% (54,762)
	Total		16% (10/62)	84% (52/62)	100% (62/62)
	κ = −0.04				

According to the SDM-Q-9-questionnaire ratings, patients felt moderately involved in decision making. We could not detect a difference (*p* = 0.8) between patients with an agenda (mean: 50 of 100 points ± 24 SD) or without expectations (mean: 49 of 100 points ± 24 SD). There were no significant correlations between participation preferences, patients' self-rated active engagement in decision making, SDM as rated with the OPTION Scale and the SDM-Q-9 (Table 4).

**Table 4:** Correlation between different aspects of decision making.

		**Agenda**	**API**	**PatPart19**	**OPTION**	**SDMQ9**	**Satisfaction**	**Patients' perceived involvement**	**Who made decisions?**	**Duration**	**MacArthur admission experience survey**
Agenda	Correlation	1	−0.20	0.30^*^	-0.03	0.03	0.06	−0.03	0.03	0.09	−0.04
	*p* =		0.16	0.03	0.87	0.85	0.68	0.84	0.81	0.55	0.77
API	Correlation	−0.20	1	−0.11	0.14	0.02	−0.19	−0.04	−0.15	−0.01	−0.15
	*p* =	0.16		0.46	0.37	0.88	0.18	0.77	0.28	0.96	0.31
PatPart19	Correlation	0.30^*^	−0.11	1	0.10	0.05	0.31^*^	0.25	−0.06	−0.11	−0.04
	*p* =	0.03	0.46		0.53	0.72	0.03	0.08	0.65	0.47	0.79
OPTION	Correlation	−0.03	0.14	0.10	1	0.16	0.10	0.06	0.19	0.31^*^	−0.22
	*p* =	0.87	0.37	0.53		0.30	0.50	0.72	0.23	0.04	0.15
SDMQ9	Correlation	0.03	0.02	0.05	0.16	1	0.41^**^	0.45^**^	−0.19	0.01	−0.36^**^
	*p* =	0.85	0.88	0.72	0.30		< 0.001	< 0.001	0.18	0.93	0.01
Satisfaction	Correlation	0.06	−0.19	0.31^*^	0.103	0.41^**^	1	0.61^**^	−0.19	−0.26	0.03
	*p* =	0.68	0.18	0.03	0.50	< 0.001		< 0.001	0.16	0.08	0.83
Patients' perceived involvement	Correlation	−0.03	−0.04	0.25	0.06	0.45^**^	0.61^**^	1	−0.29^*^	−0.31^*^	0.08
	*p* =	0.84	0.77	0.08	0.72	< 0.001	< 0.001		0.04	0.04	0.57
Who made decisions?	Correlation	0.03	−0.15	−0.06	0.19	−0.19	−0.19	−0.29^*^	1	0.39^*^	−0.01
	*p* =	0.81	0.28	0.65	0.23	0.18	0.18	0.04		0.01	0.92
Duration	Correlation	0.09	−0.01	−0.11	0.31^*^	0.01	−0.26	−0.31^*^	0.39^*^	1	−0.19
	*p* =	0.55	0.96	0.47	0.04	0.93	0.08	0.04	0.01		0.20
MacArthur admission experience survey	Correlation	−0.04	−0.15	−0.04	-0.22	−0.36^*^	0.03	0.08	−0.01	−0.19	1
	*p* =	0.77	0.31	0.79	0.15	0.01	0.83	0.57	0.92	0.20	

* *Significant*;

** *highly significant*.

Overall, patients were rather satisfied with ward rounds: 36% were very satisfied, 39% satisfied, 18% average, 7% dissatisfied and 2% very dissatisfied. There was a positive correlation between satisfaction and the SDM-Q-9-questionnaire (*p* < 0.01), perceived amount of information (*p* < 0.01), patients' perceived involvement (*p* < 0.01), the PatPart19-Questionnaire (*p* = 0.03), indicating a higher satisfaction if patients thought they were well-involved. Also, patients with high self-rated engagement in ward rounds (PatPart19 > 50) were significantly more satisfied (*p* = 0.03). There was a negative correlation between the MacArthur Admission Experience Survey and the SDM-Q-9-questionnaire, indicating a lower level of perceived participation in patients who were hospitalized against their will (*p* < 0.01). We could not detect a correlation between satisfaction and the question “who made the decision” from the patients' view. Also, there was no correlation between satisfaction and the PatPart19, API, OPTION scale and the expectation before ward rounds. Interestingly, there was a positive correlation of the OPTION scale and the duration of the ward round (Table 4).

Seventy percent of the patients considered ward rounds an important component of in-patient treatment. Patients with a clear agenda thought so significantly more frequently than patients with no expectation (*p* = 0.01).

## Discussion

In our study we identified two main prototypes of patients when it comes to ward rounds. Those who have clear expectations toward the ward round (and an agenda) and those who have not. Patients who had a clear agenda made more decisions, discussed more topics, generally felt more engaged in decision making and were more confident with the decision making process than patients with no expectations. In addition, patient participation generally was rather low with patients' perception being more positive than observer ratings. Doctors' and patients' impression of patient involvement and their role in decision making differed widely. Furthermore, patient satisfaction correlated with patients' subjectively perceived participation and involvement, whereas objectively the only positive correlation we detected concerning patient involvement was with the duration of the ward round.

### Comparison With Existing Literature

Research on psychiatric ward rounds is rare. However, as cited above, ward rounds are an essential component of psychiatric in-patient treatment, especially with regard to multidisciplinary team organization (10, 22), even if the structure of ward rounds and the people involved vary considerably (as in our study). The mean number of participants in the ward rounds was comparable to Labib and Brownells (13) findings, whereas the duration of the single consultations was considerably lower.

The main focus of our study was, however, the patients' view on ward rounds. Previous surveys have reported that many patients would prefer not to be seen at ward rounds (23), with the number of unknown team members being one of the most important reasons. The number of patients in our sample having negative expectations toward the ward round was relatively low (5%).

Our study adds more detailed data on decision making processes and the patients' perception of these processes. One of our main findings was that patients with and without an agenda differ considerably with regard to their perceptions of the ward round but also with regard to the number of decisions made. Our results suppose that coming to the ward round with an agenda might be better in various ways than having no agenda. This finding fits well with previous research addressing doctor-patient-interaction outside ward rounds (24, 25) where positive effects of patient preparation for consultations had been shown. Our study adds that these patterns might be similarly relevant forward rounds.

In terms of the general perception of SDM during ward rounds, a rather paternalistic style was observed. Surprisingly, patients tend to perceive the situation differently and feel sufficiently involved and vastly satisfied. The phenomenon of this discrepancy between objective and subjective ratings of SDM has already been shown in preceding studies (26). A possible explanation could be the ritualized character of ward rounds. The amount of attention given to the patient inherent in the occasion already evokes the feeling of being involved in the process of decision making. While indeed, beside from being heard and having the opportunity to express own wishes and needs, in most cases, no actual SDM takes place. In fact, one could argue that the mean duration of 6 minutes per conversation is surprisingly low for a crucial weekly meeting where key decisions are made and potentially the time taken is limiting patient engagement.

A limitation of our study may be a general lack in possible methods to measure shared decision making. Furthermore, many patients we approached denied to take part in the investigation due to skepticism toward clinical studies and audio recordings, which might have led to a selection bias. Also, the method we chose already changed the usual character of ward rounds, since an additional person took part and recorded the situation.

Consequently, motivating patients to have an agenda toward ward rounds should be part of the weekly routine in in-patient treatment, as well as improving patient participation and information procedures during ward rounds.

## Data Availability Statement

The raw data supporting the conclusions of this article will be made available by the authors, without undue reservation.

## Ethics Statement

The studies involving human participants were reviewed and approved by Ethics Commission of Technical University of Munich. The patients/participants provided their written informed consent to participate in this study.

## Author Contributions

FH, JH, and SH contributed to conception, design of the study, and wrote sections of the manuscript. FH organized the database and wrote the first draft of the manuscript. FH, FS, and JH performed the statistical analysis. All authors contributed to manuscript revision, read, and approved the submitted version.

## Conflict of Interest

The authors declare that the research was conducted in the absence of any commercial or financial relationships that could be construed as a potential conflict of interest.

## Publisher's Note

All claims expressed in this article are solely those of the authors and do not necessarily represent those of their affiliated organizations, or those of the publisher, the editors and the reviewers. Any product that may be evaluated in this article, or claim that may be made by its manufacturer, is not guaranteed or endorsed by the publisher.
